# Insulin Inclusion into a Tragacanth Hydrogel: An Oral Delivery System for Insulin

**DOI:** 10.3390/ma11010079

**Published:** 2018-01-05

**Authors:** Mokhamad Nur, Todor Vasiljevic

**Affiliations:** 1 Advanced Food Systems Research Unit, College of Health and Biomedicine, Victoria University, PO Box 14428, Melbourne 8001, Australia; mokhamad.nur@live.vu.edu.au; 2 Department of Agricultural Product Technology, Faculty of Agricultural Technology, Brawijaya University, Jl. Veteran, Malang 65145, Indonesia

**Keywords:** insulin, protein, peptides, PEC, hydrogels, gum tragacanth, insulin carrier, rheology, drug delivery, biopolymers

## Abstract

Nanoparticles or microparticles created by physical complexation between two polyelectrolytes may have a prospective use as an excipient for oral insulin administration. Natural polymers such as tragacanth, alginate, dextran, pullulan, hyaluronic acid, gelatin and chitosan can be potential candidates for this purpose. In this research, insulin particles were prepared by the inclusion of insulin into a tragacanth hydrogel. The effect of the pH and concentration relationship involving polyelectrolytes offering individual particle size and zeta potential was assessed by zetasizer and scanning electron microscopy (SEM). Insulin–tragacanth interactions at varying pH (3.7, 4.3, 4.6, or 6), and concentration (0.1%, 0.5%, or 1% w/w) were evaluated by differential scanning calorimetry (DSC) and ATR Fourier transform infrared (ATR-FTIR) analysis. Individual and smaller particles, approximately 800 nm, were acquired at pH 4.6 with 0.5% of tragacanth. The acid gelation test indicated that insulin could be entrapped in the physical hydrogel of tragacanth. DSC thermograms of insulin–tragacanth showed shifts on the same unloaded tragacanth peaks and suggested polyelectrolyte–protein interactions at a pH close to 4.3–4.6. FTIR spectra of tragacanth–insulin complexes exhibited amide absorption bands featuring in the protein spectra and revealed the creation of a new chemical substance.

## 1. Introduction

Development of an appropriate carrier system for the oral delivery of insulin is still the main related problem due to compromised bioavailability hindered by the epithelial barriers of the stomach and gastrointestinal destruction by proteolytic enzymes [1,2,3]. Thus, a suitable insulin carrier really should provide biocompatibility as well as stabilisation under conditions in the gut in order to assure that the primary fraction of the insulin would be biologically active when delivered on site [1,2,3].

Biopolymers, for example, chitosan, dextran sulphate, and alginates, have been extensively studied due to their suitability for encapsulating proteins/peptides [4,5]. However, after encapsulation with these biopolymers, the bioavailability of insulin after oral administration remained low. These biopolymers can be complexed with insulin using various strategies, which include polyelectrolyte complexation (PEC), emulsification, ionotropic pregelation, and spray drying. Particles formed through polyelectrolyte complexation have demonstrated potential for use as an oral insulin carrier. PEC is generally created as soon as negatively and positively charged polyelectrolytes are mixed together through electrostatic attractions [5]. To be able to form a complex, the two polymers need to be ionised as well as carrying opposing charges. Therefore, the reaction could directly take place within pH values in the area of (typically) the pKa interval of the two polymers. At the time of complexation, electrolytes may sometimes form a hydrogel [6]. However, in the case that the ionic attraction is very strong, precipitation could occur and prevent hydrogel creation. Precipitation could be prevented when electrostatic interaction is destabilised through enhancement of the ionic strength, for example, by adding salts (NaCl). Salts can diminish the interactions between positively and negatively charged electrolytes just by adding to the counter-ion environment. Therefore, basically no phase separation occurs, and a viscous and macroscopically homogeneous mix that could create a gel at a low temperature is also acquired [6].

Numerous studies have assessed the feasibility of various PECs for the delivery of insulin including alginate–chitosan [7,8], chitosan–poly(*γ*-glutamic acid) [9], and chitosan–arabic gum systems [10]. The benefit of these hydrogel systems is that the drug can be encapsulated easily by creating a water-based ionotropic gel, which protects the bioactive structure of insulin [11]. One of the main factors affecting functional properties of insulin–biopolymer complexes is pH. In alginate–insulin systems, if the environment pH is reduced from 6 to 4, the insulin encapsulation efficiency of the complexes increases [12]. This is also in accord with other research which showed that the encapsulation efficiency of insulin was around 93% [13] and 97% [14]. A possible explanation for the observed difference may be attributed to the environmental pH since it was under the isoelectric point of insulin (pI = 5.3), which gave preference to alginate–protein electrostatic attraction [15]. In another study, involving the dextran sulphate–insulin complexation, the protection has been attributed to the ionic interaction between the amino acid residues in the insulin molecules and the sulphate groups in the dextran sulphate [11]. This mechanism has also been studied when the complexation of polyion and protein decreased the release of the protein [16]. The glycemic effect was prolonged with the promotion of sustained insulin availability in vivo when dextran sulphate was included as a physical mixture in the delivery systems composed of chitosan and/or polyethylenimine [11,17].

To promote insulin absorption in the intestinal area, the PEC needs to adhere to the gastrointestinal lining at the site of delivery. Therefore, polymers with enhanced mucoadhesive properties are selected [18,19]. These types of mucoadhesive particles have the ability to extend the residence time around the site of release, trigger contact with the intestinal barrier, and produce a drug concentration gradient, which stimulates the transmission of the insulin via the intestinal membrane layer [18,20]. Recently, we reported that tragacanth gum (TG) could be used as a new polymer to deliver proteins and peptides. It is highly acid-resistant with high mucoadhesive properties [18]. Tragacanth offers special functional properties since it is safe, nontoxic, biocompatible, biodegradable, and stable over a broad range of pH [21]. Moreover, it is the most effective natural emulsifier intended for low pH O/W emulsions [21]. TG offers distinct surface activity attributes and decreases water surface tension efficiently even at very low quantities—lower than 0.25%—as well as encouraging emulsification. The zeta potential of tragacanth is about −21 mV. This can be related to the carboxylic groups of galacturonic acid (negatively charged) which is the primary chain of tragacanthin (water-soluble fraction of TG). TG is a viscous, odour-free biopolymer primarily containing two components: tragacanthin (water-soluble) and bassorin (swellable). The ratio between the soluble and insoluble fractions of TG gum in water differs considerably and depends on various Astragalus species [21]. Interaction with other material via hydrogen bonding and crosslinking can be initiated by using these TG functional groups (i.e., carboxylic acid and hydroxyl) [21].

The TG polymer has the capacity to create a gel via the carboxylic groups of tragacanth. Therefore, tragacanth provides a possibility to create an interaction with insulin, particularly in an acidic environment (under the pI of insulin) [18]. In other research, TG was applied in quercetin encapsulation through structuring of the TG shell and polycaprolactone (PCL) core self-assembled micelles. The quercetin release from these micelles showed a pH dependence. The rate of release was increased considerably at pH 2.2 [21].

A gelation and mucoadhesion study indicated that tragacanth has the potential to become an excipient for the oral administration of protein/peptides, for instance, insulin [18]. It is conceivable that tragacanth properties may enhance the drug loading capacity, encapsulation efficiency, and the stability of the insulin encapsulated in the tragacanth particles through ionic attraction between tragacanth and the amino groups of the amino acid residues in insulin. Herein, we report our result on the use of tragacanth as an alternative choice and enhanced carrier for the oral administration of insulin. The approach was designed to monitor the complexation of the polyelectrolyte in becoming an insulin excipient. Systems produced from the complexation of polyelectrolytes at different pH and stoichiometric relationships involving polyelectrolytes were freeze-dried and/or directly analysed to verify interactions between tragacanth and insulin.

## 2. Materials and Methods

### 2.1. Materials

Tragacanth was obtained from Sigma–Aldrich (Castle Hill, NSW, Australia). GDL (Glucono-δ-lactone) powder from Sigma–Aldrich was also applied with no additional purification. The insulin sample that contains 100 U/mL of insulin was obtained from Novo Nordisk A/S (Bagsvaerd, Denmark). The water utilised was of a Millipore level of quality.

### 2.2. Characterisation

#### 2.2.1. Microparticle Preparation

TG stock solution (2% w/w) was well prepared by dissolving a proper amount of the powder in MilliQ water at different pH levels (3.7; 4.3; 4.6; or 6), which was adjusted by adding an appropriate quantity of glucono δ lactone (GDL) powder. GDL dissociates in water, releasing gluconic acid and lowering the pH of the solution. The advantage of this type of pH adjustment is that the change is achieved at a slower rate without any change in the bulk volume. Sodium azide was included throughout the preparation of samples (0.2 g/L) to prevent microbial growth. The resulting solution was gently stirred with a magnetic rod at room temperature and kept overnight at 4 °C.

Tragacanth and insulin microparticles were prepared via mixing insulin (0.2 mg/mL) and TG colloidal solutions containing a different concentration of tragacanth (0.1%, 0.5%, or 1% w/w) at a predetermined pH. The complexation was allowed to proceed overnight by gently stirring the solution with a magnetic rod at room temperature. The mixture was then centrifuged at 20,000× *g* for 60 min at a room temperature using a high-performance centrifuge (Beckman Coulter Inc., Brea, CA, USA). The sedimented PECs were then frozen at −20 °C and freeze-dried at 0 °C for at least 48 h using a freeze-drier (model FD-300, Airvac Engineering Pty. Ltd., Dandenong, Australia).

#### 2.2.2. Acid-Induced Gelation

GDL-induced acidification was carried out to evaluate the capability of TG to entrap insulin through acid-induced gelation [18]. Dynamic small amplitude oscillatory analysis was carried out using a stress-controlled rheometer (MCR 301, Anton Paar GmbH, Ostfildern, Germany) with a double gap geometry (DG 26.7-SN. 24845, Anton Paar) to determine an acid gel point following a previously described method [18]. The required amount of the GDL powder was added to a TG solution at 20 °C. An exact volume of the sample (3.9 mL) was added directly into the testing system (double gap) at the same temperature and allowed to stabilize for 100 min (time sweep) during which time changes in the viscoelastic behaviour of the colloidal solution were recorded. The change of pH was concurrently noted every 2.5 min in the remaining part of the sample by using a pH meter (Model 8417; Hanna Instruments, Singapore) during the same period.

#### 2.2.3. Particle Size and Zeta Potential Analysis

The particle size and zeta potential of the polymeric complexes created by mixing the insulin solution (0.2 mg/mL) and the TG solution (0.1%, 0.5%, or 1% w/w tragacanth) at different pH (3.7, 4.3, 4.6, or 6) were analysed using a zetasizer (ZEN3600, Malvern Instrument Ltd., Worcestershire, UK) with a He–Ne laser beam at 658 nm. An appropriate aliquot of the insulin–tragacanth mixture was diluted 1:100 with MilliQ and stored overnight before the measurement [18].

#### 2.2.4. Measurement of Loading Efficiency

Loading efficiency was determined indirectly following centrifugation of the insulin and TG dispersion upon mixing insulin (0.1 mg/mL) and TG colloidal solutions at different pH and tragacanth concentrations. The PEC was centrifuged at 20,000× *g* for 60 min at room temperature using a high-performance centrifuge (Beckman Coulter, Brea, CA, USA). The quantity of insulin in the supernatant was analysed using the Bradford procedure at 595 nm [18]. The loading efficiency was measured as

(1) $$\mathrm{Loading}\;\mathrm{efficiency}\;\left(\%\right)=\frac{\mathrm{Total}\;\mathrm{amount}\;\mathrm{of}\;\mathrm{insulin}-\mathrm{Free}\;\mathrm{insulin}\;\mathrm{in}\;\mathrm{supernatant}}{\mathrm{Total}\;\mathrm{amount}\;\mathrm{of}\;\mathrm{insulin}}\times 100.$$

#### 2.2.5. DSC (Differential Scanning Calorimetry) Analysis

Thermal characteristics of particles were analysed using DSC, as explained earlier [15], with some adjustments. Thermograms of TG solutions were gained by using a DSC (Mettler Toledo, Schwerzenbach, Switzerland) fitted with an intracooler system and under an inert nitrogen gas atmosphere. A sample (4–7 mg) obtained under described experimental conditions (variable pH and tragacanth concentration) was put in a 40 µL aluminium lightweight pan, hermetically enclosed just before insertion into the DSC, and then heated from 20 to 350 °C at a constant rate of 10 °C/min within constant purging of nitrogen at 20 mL/min. An empty pan with the same weight functioned as the reference. The ΔH values, onset, endset, and peak temperatures of the thermograms were documented.

#### 2.2.6. FTIR Analysis

FTIR spectra of the particles at different pH (3.7, 4.3, 4.6, 6) and concentrations of tragacanth (0.1%, 0.5%, and 1%) were acquired by using a Perkin Elmer ATR-FTIR spectrometer equipped with a Diamond TM ZnSe single reflection ATR plate (Perkin-Elmer, Norwalk, CT, USA). The actual spectrum of every sample was acquired from 16 scans from 600 to 4000 cm^−1^ having a resolution of 4 cm^−1^ as well as strong apodisation. This particular measurement was adjusted towards the background spectrum of the solvent. Baseline manipulation and data acquisition were gained using Shimadzu IR solution software v1.40 [18,22].

#### 2.2.7. Scanning Electron Microscope (SEM)

Particle morphology was studied using scanning electron microscopy (SEM). For SEM analysis, samples of microparticulate complexes were installed on metal stubs, gold covered under vacuums and then analysed in a JEOL NeoScope JCM-5000 A SEM (10 kV, Tokyo, Japan).

### 2.3. Statistics

The information acquired from particle size analysis was arranged in a randomised block design using pH as the primary factor. These assessments were duplicated at least once, with subsequent subsampling providing a number of independent observations of at least *n* ≥ 4. Final results were analysed using one-way ANOVA, SAS (1996). Tukey’s Studentized Range (HSD) analysis was applied for multiple comparisons of means. The degree of significance was predetermined at *p* = 0.05.

## 3. Result and Discussion

The application of a biopolymer as a multiparticulate excipient for protein/peptide delivery has long been extensively recorded in the scientific literature [2,23]. This kind of matrix currently has great potential to be applied for the controlled release of drugs because of its relatively small molecular size. Furthermore, bioavailability and drug absorption could be improved as a result of a large surface area to volume level ratio, which leads to significantly more intimate contact along with the mucus layer [11,23].

In this study, initially, microparticulate polyelectrolyte complexes between insulin and tragacanth were created, including the mild mixing of two aqueous solutions of tragacanth and insulin. To test the ability to create a gel, GDL was added to the mixture, and an acid gelation test was conducted.

**Figure 1:**
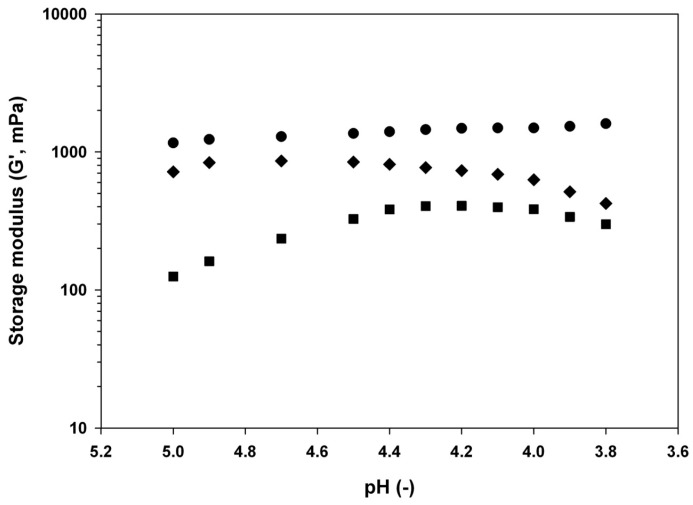
Evolution of storage modulus (G′) during acid-induced gelation of tragacanth dispersions at ◆ 0.1% w/w, ■ 0.5% w/w, and ● 1% w/w. Measurements were performed at 20 °C at constant strain (1%) and frequency (1 Hz).

It can be seen from Figure 1 that during the acid gelation test, as the pH was decreasing, at pH around 4.3, the storage modulus was greater than that at other pH levels. Almost all of the systems with viscoelastic properties possess both viscous (liquid) and elastic (solid) elements, in which the shear stress is between 0 and 90 degrees. In these systems, the stress element that is in-phase with the shear strain is in charge of the elastic element and is identified as the storage modulus (G′), which depicts the material elasticity. The value of the storage modulus is proportional to the quantity of permanent interactions and the strength of the interactions existing in the biopolymers. Therefore, G′ is a measure of the structure of the biopolymers [24]. A time sweep offers the viscoelastic properties of biopolymers as a function of time, in which the strain, frequency, and temperature are kept constant. The gel networks of biopolymers continue to develop throughout a time sweep. This can be noticed from the increase in the value of the storage modulus as a function of time [24]. In our system, during the time sweep, a change of pH was measured (as described in Section 2.2.2). Therefore, a storage modulus vs pH graph was created. The increase in the storage modulus (maximum at pH 4.3), which is close to the gelling point of tragacanth and insulin [18], was an indication that insulin was likely entrapped in the tragacanth gel (hydrogel creation). Carboxylic groups from tragacanth may be involved in this process. Most of the pH-sensitive biopolymers consist of pendent basic or acidic groups, which either take or give protons in reaction to the solvent pH [25]. Polyacid biopolymers are unswollen in an acidic environment since their acid groups are unionised and protonated [25]. Upon increasing the pH, a negatively charged polymer would swell. The opposing patterns are noticed in polybasic biopolymers, considering that the ionisation of the basic groups increases the following decline in pH [26]. Some instances of pH-sensitive biopolymers having anionic groups are polycarboxylic acids (PAA) or poly-methacrylic acid (PMA) and poly-sulfonamides (derivatives of p-aminobenzene sulfonamide). In an acidic environment, hydrophobic interactions dominate and carboxyl groups are protonated, resulting in volume withdrawal involving the biopolymer consisting of carboxyl groups. In a basic environment, carboxyl groups dissociate into carboxylate ions, causing higher charge density in the biopolymer, resulting in swelling. The chain configuration of a weak polyacid is a function of the pKa of the biopolymer [27].

This type of gel is called a physical gel due to the fact that the networks tend to be retained by molecular entanglements and/or weak forces including hydrophobic, H-bonding, or ionic interactions [28]. The network porosity of such hydrogels changes along with electrostatic repulsion. An ionic gel consisting of carboxylic and/or sulfonic acid groups demonstrates either immediate or slow changes in their particular dynamic or equilibrium swelling behaviour due to the change in the environmental pH. The ionisation degree of those hydrogels is determined by a number of pendant acidic groups within the gel, causing enhanced electrostatic repulsions involving negatively charged carboxyl groups at various chains. This, consequently, leads to increasing hydrophilicity of the network as well as a higher swelling ratio within a higher pH [28]. On the other hand, a hydrogel consisting of basic pendant groups, including amines, will ionise as well as demonstrate electrostatic repulsion in an acidic environment [28].

It can also be noticed from Figure 1 that tragacanth at a higher concentration (1%) exhibited stronger viscoelastic properties than at the lower concentrations (0.5% and 0.1%). If this is linked to pI, the achievable entrapment of a protein and/or a peptide by TG is most likely achieved under the isoelectric point (pI) of insulin [18]. For instance, the pI of insulin can vary from 5.5 to 6.4, based on its origin. At a pH higher than its pI value, insulin will be mainly negatively charged [29]. This insulin characteristic could be utilised to create insulin–biopolymer complexes through electrostatic attraction with tragacanth. At pH around 4.3 and 4.6, TG may undergo coacervation with insulin as well as simultaneously creating a hydrogel which is able to entrap insulin since, at this pH, insulin can be positively charged close to the determined gelling point of TG [18,30].

**Figure 2:**
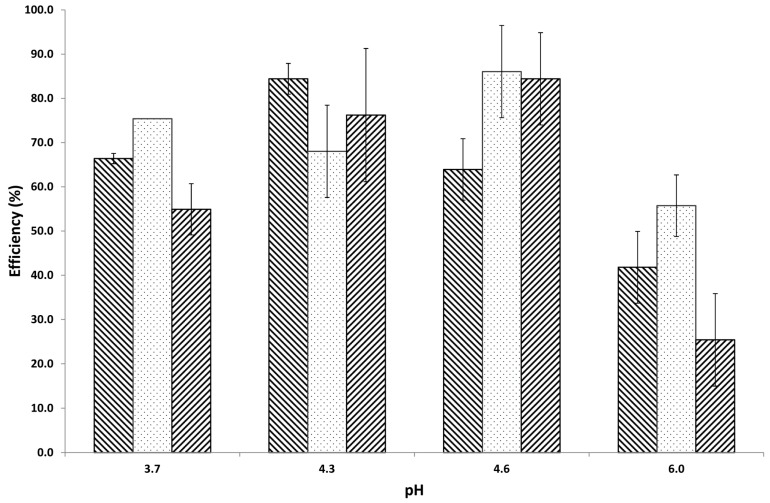
Loading Efficiency (LE) of insulin complexing with tragacanth at different concentrations (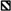 −0.1% w/w, 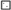 −0.5% w/w, and 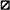 −1% w/w) at different pH (3.7, 4.3, 4.6, or 6) adjusted by addition of GDL at room temperature and equilibration under very low magnetic stirring overnight.

After a mixture of a gel-like formation was created, it was then freeze-dried, and a loading efficiency (LE) was examined at different levels of pH and concentrations. The pH variety was selected to obtain opposite charges of electrolytes as well as suitable complex creation. Within this particular pH range, electrostatic attraction involving proteins and biopolymers occurs. It can be observed from Figure 2 that the reduction of pH from 6 to 3.7 resulted in an increase of LE, especially at pH 4.6. However, if the pH of the aqueous solution was set to 3.7 or 4.2, the particles become much larger (>800 nm). In this pH range, some parts of tragacanth begin to precipitate [31], which may play a role in the increased mean particle size (Table 1). Therefore, insulin was partly bound ionically to the insoluble uronic acid of tragacanth. The interaction involving biopolymers and insulin is mostly ionic. However, one should also consider hydrogen bonding as well as van der Waals forces [32,33]. It can be seen from Table 2 that negative zeta potential (ZP) values are acquired because of the carboxylic groups [34] of tragacanth [18]. Moreover, the ZP values depend on the dispersion pH [34]. In general, if the ZP values are less than −10 mV (in most cases, from −25 to −30 mV, Table 2), it can predict an excellent colloidal stability because of the high energy barrier between particles [34]. Positive amino radicals of insulin are highly and electrostatically interacted with by carboxylic/sulphate groups. At pH 4.3 or 4.6, insulin is primarily positively charged (pI of insulin: 5.3) [35] and is therefore attracted to the partly negative tragacanth, while at pH 6, positive charges are minimised on the amino acid, which could prevent attractive interactions with the negative charges on the tragacanth. As a result, the insulin LE is less at pH 6 than at other, lower pH. For that reason, pH 4.6—at which microparticles were produced, and a high insulin LE was acquired—was selected as the most appropriate pH. The outcomes acquired suggested that the affinity of insulin for tragacanth carboxylic groups is greatest at pH 4.3 or 4.6, as pointed out by examining the LEs of the created complexes. A tragacanth concentration of 0.5% (w/w) tends to be the optimum concentration for complexation. Particularly, at pH 4.3, the LE increased from 65% (0.1%, w/w of TG) to 89% for TG concentration 0.5% (w/w).

Insulin and biopolymer complexes could be produced at the isoelectric point (pI) of insulin. The pI of insulin is around 5.5–6.4. At a pH under the pI value, insulin is positively charged; the converse is also true [2]. These attributes of insulin have been used in the creation of insulin–biopolymer complexes through electrostatic interaction with a negatively charged biopolymer (alginate) as well as positively charged biopolymer (chitosan), which are protonated at pH values under its pKa (6.5) [2]. TG has the propensity to form a complex with insulin at a pH under the pI of insulin [18]. Conversely, a positively charged biopolymer such as chitosan can form a complex with insulin if the pH is altered over the pI of insulin [36,37].

**Figure 3:**
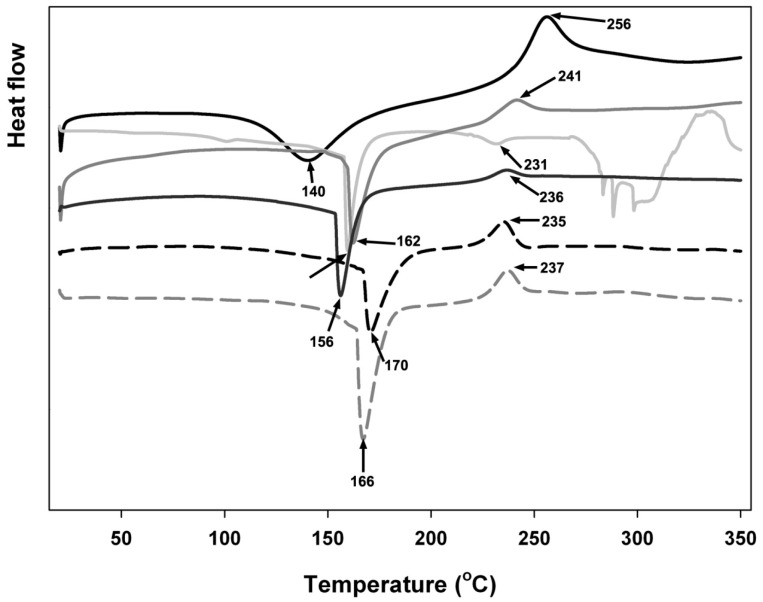
DSC thermograms of polymeric complexes created by mixing insulin solution (0.1 mg/mL) and tragacanth solution at 0.1%, 0.5%, or 1% w/w concentration at different pH (3.7, 4.3, 4.6, or 6) adjusted by addition of GDL at room temperature and equilibration under very low magnetic stirring overnight. Legend: 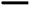 tragacanth (control); 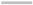 insulin (control); 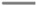 mixture at pH 3.7; 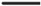 mixture at pH 4.3; 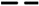 mixture at pH 4.6; and, 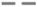 mixture at pH 6.0. Arrows and numbers indicate the temperature of phase transition.

DSC thermograms in Figure 3 show variations involving individual biopolymers and, after complexation, suggest ionic interactions indicated by the change of endothermic peaks as well as by the shift in exothermic peaks associated with decomposition temperature. The DSC curves exhibit a wide endothermic peak between 100 and 200 °C for isolated polyelectrolyte and its physical mixture. All samples exhibited exothermic peaks between 220 and 285 °C.

Individual biopolymers were recognised by the occurrence of first endothermic peaks at 140 °C for tragacanth only and 156 °C, 162 °C, 166 °C, 170 °C for the mixtures at different pH, respectively, and also by the occurrence of greater exothermic peaks at 256 °C for tragacanth only and 235 °C, 336 °C, 237 °C, 241 °C for the mixtures at different pH, respectively. Exothermic peaks are correlated with the destruction of polyelectrolytes as a result of dehydration as well as depolymerisation reactions, most likely due to the incomplete decarboxylation of the protonated carboxylic groups as well as oxidation reactions of the polyelectrolytes, while endothermic peaks are related to the reduction of water connected to the hydrophilic groups of the biopolymers [38,39,40].

Exothermic and endothermic peaks shifted to greater temperatures when the pH of the microparticles was reduced from 6 to 4.3. It was noticed that lowering the pH resulted in greater stability of the microparticle; therefore, more energy was required in order to eliminate residual water adsorbed onto the mixture (endothermic peak changed to increased value), and a lesser amount of energy was discharged by breaking ionic attractions and through microparticle thermal decomposition (exothermic peak change to increased value) [41].

Like alginate and pectin, TG possesses a carboxyl group (galacturonic acid). Consequently, the charge variations in TG are caused by this particular carboxyl group. The most typical negatively charged side groups on polysaccharides are usually sulphate groups or carboxylate groups. The negative charge of agars and carrageenans comes from sulphate groups, but pectins and alginates obtain their negative charges from carboxylate groups [42]. TG has a previously documented pKa value of 3 [18].

The optimum ionic attraction between tragacanth and insulin was achieved at lower pH. Almost similar to alginate, tragacanth gel shrunk at lower pH levels due to a decrease in the pore size of the tragacanth matrix [18,43]. For that reason, at pH 4.3 or 4.6 it is feasible that microparticles introduced a more powerful robustness than at pH 6. Interactions between tragacanth and insulin have been identified to become pH-dependent together with more powerful complexes that were previously acquired at pH close to 4.2–4.7 [18].

The inclusion of insulin within microparticulate complexes can certainly be seen by the postponing of its endothermic peak. The two endothermic peaks in connection with insulin, which are related to water loss and the denaturation process [42], started to be indistinct and changed themselves into a single peak following entrapment in the microparticulate complexes. Insulin-loaded models achieved this particular endothermic peak at a lower temperature in comparison to insulin-free models, apparently showing an attraction involving the protein and the tragacanth. Furthermore, looking at the exothermic conditions of insulin-loaded and unloaded particles, the onset point began at a lower temperature for insulin-loaded microcomplexes; this probably suggests that entrapped insulin initiated decomposition at higher temperatures (235–241 °C) than when analysed in isolation from the particles (231 °C).

**Figure 4:**
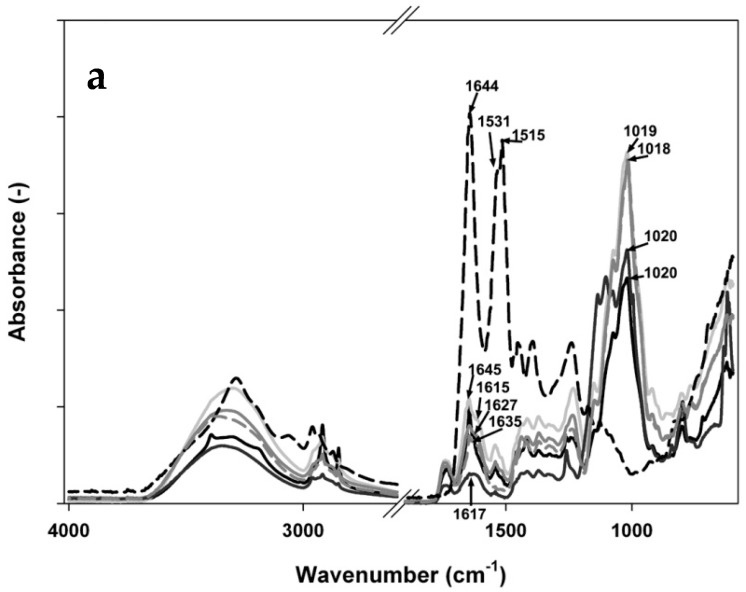

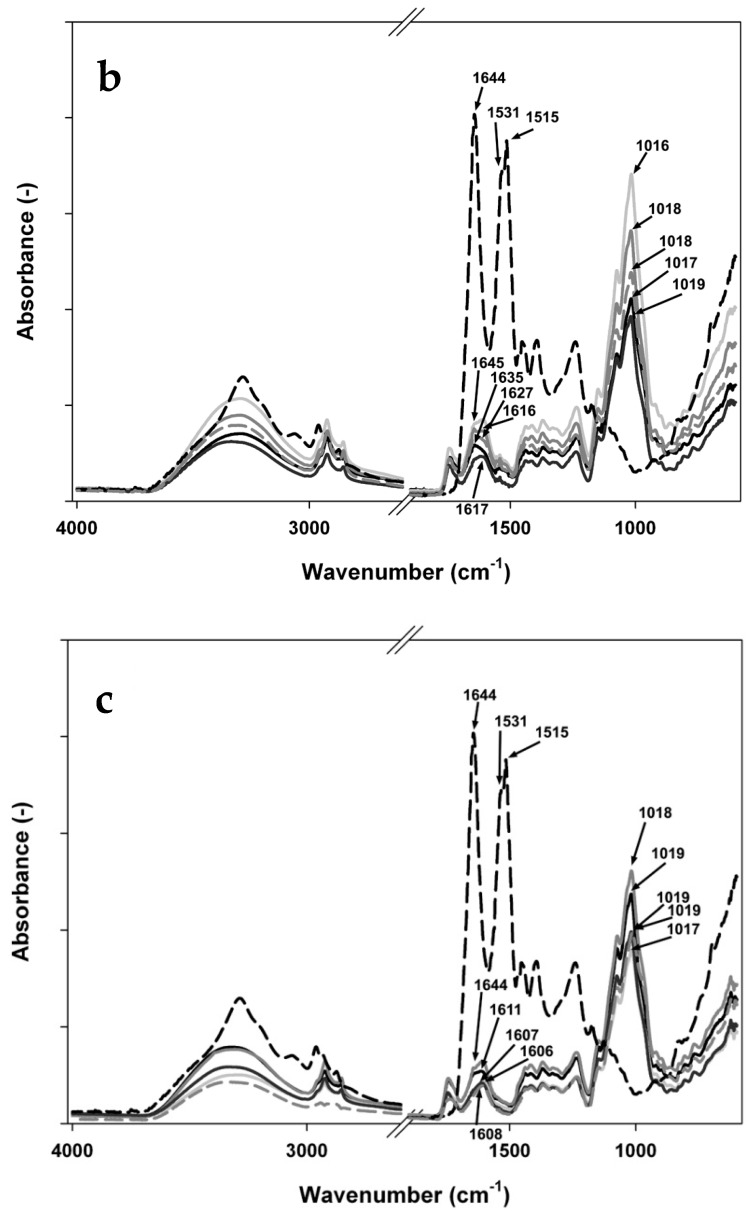
FTIR spectra of polymeric complexes created by mixing insulin solution (0.1 mg/mL) and tragacanth solution at (**a**) 0.1%, (**b**) 0.5%, or (**c**) 1% w/w concentration at different pH (3.7, 4.3, 4.6, or 6) adjusted by addition of GDL at room temperature and equilibration under very low magnetic stirring overnight. Legend: 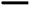 tragacanth (control); 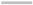 insulin (control); 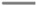 mixture at pH 3.7; 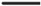 mixture at pH 4.3; 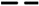 mixture at pH 4.6; and, 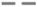 mixture at pH 6.0. Arrows and numbers indicate a wavenumber of a particular structural change.

The obtained FTIR spectra are represented in Figure 4a–c, and show two shoulders on the complex absorption bands in Amide I (∼1644 cm^−1^) as well as in Amide II (∼1531 cm^−1^) that are properties of the protein spectra. The monomer of insulin has numerous ionizable groups because of six amino acid residues able to attach a positive charge and ten amino acid residues able to attain a negative charge [44,45]. These kinds of characteristics are, therefore, probably responsible for the entrapment of insulin into tragacanth microparticles.

The bands at 1416 and 1369 cm^−1^ are associated with the symmetric stretching of carboxylate groups as well as the methyl groups in methyl esters of galacturonic acid, respectively, while the actual vibrational modes of COOH in galacturonic acid and its salts and esters contain asymmetric stretching (1740–1600 cm^−1^). Polygalacturonic acids possess the highest possible absorption band in this region, having quite strong absorptions at 1017 and 1020 cm^−1^, and optimum absorption bands at 1018 and 1019 cm^−1^ representing the occurrence polysaccharides which have galactose, for example, arabinogalactans and galactans [18,46].

It can be seen that tragacanth carboxyl peaks close to 1627 cm^−1^ (symmetric COO– stretching vibration) and 1416 cm^−1^ (asymmetric COO– stretching vibration) broadened and shifted a little from 1627 to 1616 cm^−1^ and 1416 to 1415 cm^−1^ following complexation with insulin. Moreover, the FTIR spectrum of tragacanth exhibits a peak associated with an amide bond at 1645 cm^−1^. Noticed shifts in the absorption bands of the carboxyl groups, amide bonds, and amino groups could be assigned to an ionic attraction between the carboxyl group of tragacanth and insulin [47]. Also, the peak absorbance of amino groups of tragacanth at 1149 cm^−1^ existed right after complexation. Similar observations were noted previously [48,49,50]. These findings indicate an effective interaction between biopolymers corresponding to the stoichiometric ratios between them, which indicate the occurrence of TG at the end of the mixture [15,51].

**Figure 5:**
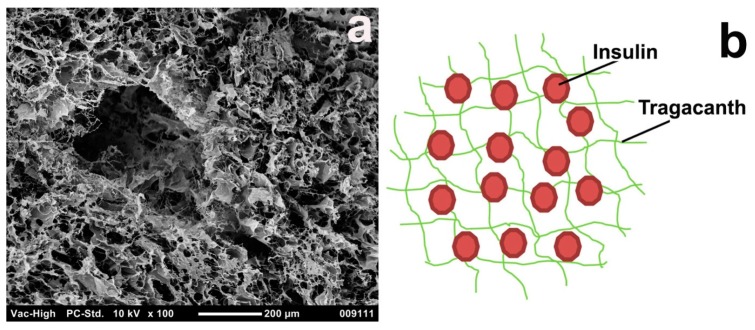
SEM image of (**a**) a complex between insulin (0.1 mg/mL) and 0.5% of tragacanth at pH 4.6 with the best exhibited efficiency and (**b**) proposed polyelectrolyte complexation (PEC) model of insulin entrapment in a tragacanth network.

Surface morphology information for freeze-dried microspheres has been acquired by SEM analysis and is presented in Figure 5a. The carrier exhibited an irregular shape and had a somewhat wrinkled surface. Apparently, the spherical shape of the microspheres was lost after drying. We speculate that insulin entrapped in tragacanth, as in Figure 5b, explains the results of SEM observation. It could be seen that, at the beginning, the tragacanth creates a homogenous network from the core to the edge. Insulin is then entrapped inside the network. The negatively charged tragacanth creates a complex with the positively charged insulin [52,53]. The microstructure (SEM) of the tragacanth hydrogels after freeze-drying exhibits the porous morphology, with pore size greater than the submicron range. The pore structure and size were similar to other acidified gel biopolymers including tragacanth-milk [54] and pectin-sodium caseinate systems [55]. These morphological characteristics are related to the exchange of insulin-loaded microparticles. The destruction of hydrogels is followed by the discharge of insulin from hydrogels. These morphological properties have been connected with water exchange and swelling of hydrogels. Swelling characteristics of hydrogels are essential for material transfer when applied to insulin carriers [56].

## 4. Conclusions

Insulin was entrapped in physical hydrogel and polyelectrolyte complexes (PECs) created using biodegradable biopolymer—tragacanth. Microparticulate complexation between tragacanth and insulin was revealed by FTIR and DSC measurement. These microparticles appear to have potential functional properties for oral insulin delivery, especially those that contain tragacanth polyelectrolytes at pH 4.3 and 4.6, although additional in vivo research should be carried out to ensure the presence of these properties.

## Figures and Tables

**Table 1:** Particle size of polymeric complexes created by mixing insulin solution (0.2 mg/mL) and tragacanth solution at 0.1%, 0.5%, or 1% w/w concentration at different pH (3.7, 4.3, 4.6, or 6) adjusted by addition of glucono δ lactone (GDL) at room temperature and equilibration under very low magnetic stirring overnight. The results are presented as means of at least five independent observations (*n* ≥ 5) with ±SE.

pH	Particle Size, nm		
	Tragacanth concentration, % w/w		
	0.1	0.5	1
3.7	1105 ± 48	1382 ± 141	839 ± 22
4.3	667 ± 37	811 ± 20	957 ± 56
4.6	651 ± 09	601 ± 19	649 ± 25
6	566 ± 23	373 ± 08	719 ± 05

**Table 2:** Zeta potential of polymeric complexes created by mixing insulin solution (0.2 mg/mL) and tragacanth solution at 0.1%, 0.5%, or 1% w/w concentration at different pH (3.7, 4.3, 4.6, or 6) adjusted by addition of GDL at room temperature and equilibration under very low magnetic stirring overnight. The results are presented as means of at least five independent observations (*n* ≥ 5) with ±SE.

pH	Zeta Potential, mV		
	Tragacanth concentration, % w/w		
	0.1	0.5	1
3.7	−22.3 ± 0.6	−26.0 ± 0.6	−29.1 ± 0.5
4.3	−38.9 ± 1.2	−19.2 ± 2.5	−30.9 ± 1.9
4.6	−36.3 ± 1.7	−7.5 ± 0.4	−18.1 ± 0.6
6	−38.1 ± 1.3	−39.5 ± 4.9	−40.8 ± 8.4
